# GATA binding protein 5 (GATA5) induces Rho GTPase activating protein 9 (ARHGAP9) to inhibit the malignant process of lung adenocarcinoma cells

**DOI:** 10.1080/21655979.2022.2025695

**Published:** 2022-01-18

**Authors:** Wenfei Ji, Lili Zhang, Hongjun Zhu

**Affiliations:** Department of Oncology, Nantong Third Peopleʹs Hospital Affiliated to Nantong University, Nantong, P.R. China

**Keywords:** Lung adenocarcinoma cells, Gata binding protein 5 (GATA5), Rho GTPase activating protein 9 (ARHGAP9)

## Abstract

Lung adenocarcinoma is the main cause of the excessive mortality for patients who lives with lung cancers. According to the GEPIA database analysis, GATA5 and ARHGAP9 were found to be low expressed in lung adenocarcinoma, and they were positively correlated, and in addition ARHGAP9 low expression was associated with poor prognosis in lung adenocarcinoma. Therefore, the present study focused on the effect of promoting GATA5 to induce ARHGAP9 on the malignant process of lung adenocarcinoma cells. The expressions of GATA5 and ARHGAP9 were measured with Western blot and RT-qPCR. With the adoption of CCK-8, EDU staining, transwell and colony formation, the cell viability, proliferation, invasion and tumorigenesis ability were detected, respectively. In addition, the wound healing and Western blot were employed to evaluate migration and metastasis-related proteins individually. Moreover, the luciferase activity as well as the binding of GATA5 and ARHGAP9 promoters were detected by luciferase report and ChIP. After further comprehensive assessments, the results confirmed that GATA5 could successfully activate ARHGAP9. Moreover, ARHGAP9 upregulation remarkably inhibited lung adenocarcinoma cell proliferation, invasion and migration as compared to the control group. More importantly, GATA5 silencing reversed the inhibitory effect of ARHGAP9 upregulation on the malignant progression of lung adenocarcinoma cells. To conclude, the present study successfully demonstrated for the first time that GATA5-induced ARHGAP9 upregulation has a protective effect on lung adenocarcinoma cells.

## Introduction

Lung cancer (LC) is the leading cause of cancer deaths (18.4% of all cancer deaths), closely followed by mortality from colorectal cancer (9.2%), gastric cancer (8.2%) and liver cancer (8.2%) [1,2]. As a member of non-small-cell lung carcinoma, lung adenocarcinoma comprises approximately 40% of all patients living with lung cancers [3]. Despite that diagnosis and treatment methods have made great progresses in the past decades, the average 5-year relative survival rate of lung cancer is only 18% [4]. Currently, the diagnosis of lung cancer mainly includes histopathological examination, cancer molecular biomarkers, imaging evaluations, whereas these methods are difficult to detect lung tumor [5,6], which may explain why lung cancer patients have high mortality. Considering this, we designed this study, aiming to further explore the mechanism of lung adenocarcinoma and develop effective methods for its prevention and treatment.

fRho GTPase activating protein 9 (ARHGAP9) belongs to Rho family of small guanosine triphosphatases (RhoGTPases) activating protein [7]. RhoGTPases act as a key player in mesenchymal transition (EMT), migration and invasion of cells [8,9]. All these cell functions have close relation with the metastasis of cancer cells and the overall survival of cancer patients [10,11]. The study held the opinion that ARHGAP9 gained a huge growth in acute myeloid leukemia patients and ARHGAP9 overexpression is related with the low overall survival rate [12]. Besides, it was also reported that ARHGAP9 overexpression resulted in poor survival of breast cancer patients while ARHGAP9 silence inhibited the proliferation, migration and invasion in breast cancer [13]. And ARHGAP9/Forkhead Box J2 (FOXJ2) inhibits cell migration and invasion during the development of hepatocellular carcinoma through induction of E-cadherin transcription [14].

GATA binding protein 5 (GATA5), a member of GATA transcription factor family, plays a critical role in the embryonic development of heart and lung [15]. Besides, as a transcription factor, GATA5 was found to suppress the progression of many human cancers [16]. Take cholangiocarcinoma as an instance, GATA5 upregulation was reported to suppress the growth, migration and invasion of cholangiocarcinoma cells [17]. Additionally, it was also reported that GATA5 inhibited hepatocellular carcinoma progression [18].

Interestingly, Analysis of the Gene Expression Profiling Interactive Analysis (GEPIA) database revealed a positive correlation between GATA5 and ARHGAP9 expression. Meanwhile, the binding site of transcription factor GATA5 and ARHGAP9 promoter was successfully predicted by JASPAR database. Therefore, this study was the first to explore the malignant process of lung adenocarcinoma cells through inducing ARHGAP9 by GATA5

## Material and methods

### Cell culture and transfection

Human bronchial epithelial cells (16HBE) and human lung cancer cells (SK-MES-1, NCl-H520, NCl-H1975 and A549) were purchased from BeNa Culture Collection (Langfang, China). Dubelccoʹs modified eagle medium (DMEM; Gibco, NY, USA) supplemented with 10% fetal bovine serum (FBS; Gibco, NY, USA), 100 U/ml penicillin and 100 µg/ml streptomycin (Invitrogen; CA, USA) was applied to culture the cells at 37°C in a humid incubator with 5% CO_2._

For transfection, siRNA-Negative Control (si-NC), overexpression plasmids-Negative Control (Oe-NC), si-GATA5 and Ov-ARHGAP9 were procured from GenePharma (Shanghai, China). With the application of Lipofectamine 2000 transfection reagent (Invitrogen, CA, USA), the cell transfection was performed.

### Western blot

Total proteins were lysed with radioimmunoprecipitation (RIPA) lysing buffer (Solarbio, Beijing, China) and quantified using Bicinchoninic acid (BCA) kit (Beyotime, Shanghai, China). Subjected to 10% gel with sodium dodecyl sulfate polyacrylamide gel electrophoresis (SDS-PAGE), the proteins were transferred onto polyvinylidene fluoride (PVDF) membranes. Subsequently, the membranes were inhibited by 5% nonfat milk for 2 h at room temperature, following which the primary antibodies against ARHGAP9, E-cadherin, MMP2, N-cadherin, Vimentin and GATA5 were utilized to incubate the membranes at 4°C overnight. On the next day, the membranes were incubated with secondary antibodies for 2 h at room temperature. Finally, the protein bands were imaged with an ECL Chemiluminescence Kit (Beyotime, Shanghai, China).

### Reverse transcription-quantitative PCR (RT-qPCR)

RNA form sample cells were extracted using TRIzol reagent TRIzol reagent and reversely transcribed into complementary DNA (cDNA) using a cDNA Synthesis Kit (Invitrogen, CA, USA). With the application of SYBR Premix Ex Taq reagents (Takara, Tokyo, Japan), real-time PCR for gene quantitation was conducted on ABI 7500 quantitative PCR instrument (Applied Biosystems, CA, USA). Glycerol-3-phosphate dehydrogenase (GAPDH) acted as the endogenous control and the relative gene expression was determined using 2^−ΔΔCt^ method. The primers used are as follows: ARHGAP9 forward 5ʹ-AACAGCCTGGTGTTCTACCG-3ʹ and reverse 5ʹ- TCCCGCTCACCCGATAAATG-3ʹ; GATA5 forward 5ʹ-CTGCCCGCTGGTCAAGA −3ʹ and reverse 5ʹ-CCGTGTCTGGATGCTTTCCT-3ʹ; GAPDH forward 5ʹ-CTGGGCTACACTGAGCACC-3ʹ and reverse 5ʹ-AAGTGGTCGTTGAGGGCAATG −3ʹ.

### Cell proliferation assay

A549 cells were inoculated into six-well plates, after which 5-ethynyl-2ʹ-deoxyuridine (EDU) solution (Beyotime, Shanghai, China) was added and the cells were incubated for 4 h. Thereafter, the digestion, centrifugation and fixation of A549 cells were conducted. With the application of 0.5% Trionx-100, the cells were permeated. After incubation with click reaction solution for 30 min in dark, the cells were photographed under a fluorescence microscope.

A549 cells that inoculated into 96-well plates were incubated for 24, 48 and 72 h. Thereafter, 10 μl CCK-8 reagent (Beyotime, Shanghai, China) was added into each well to incubate cells for another 2 h. In the premise of λ = 450 nm, a microplate reader (Bio-Rad, USA) was utilized to detect the absorbance.

### Colony formation assay

For colony formation, DMEM medium that supplemented with 10% FBS was employed to re-suspend the A549 cell suspension. The cells were inoculated into culture dishes and incubated at 37°C with 5% CO_2_. After that, the colonies were fixed with 4% paraformaldehyde and stained with 0.5% crystal violet solution, respectively. At last, the number of colonies was counted with the adoption of a Nikon Eclipse E600 microscope (Nikon Instruments, USA; magnification, x100).

### Cell migration assay

A549 cells were inoculated in 6-well plates and incubated until reached 80–90% confluency. Subsequently, a pipette tip was applied to make a wound in the cell monolayer. After washed with PBS, the cells were incubated in an incubator at 37°C containing 5% CO_2_ and recorded at 0 and 24 h. Finally, the area occupied by migrated cells was visualized using Image J software (magnification, x100).

### Cell invasion assay

Transwell was adopted to assess the invasiveness of A549 cells. The upper chamber containing 5% CO_2_ was used to culture A549 cells, at the same time, 10% FBS was added into the lower chamber. After that, the fixation and staining of cells were conducted with 4% paraformaldehyde and 0.1% crystal violet. At last, the images of cell invasion were observed under a microscope (magnification, x100).

### Luciferase report gene assay

GEPIA database (http://gepia.cancer-pku.cn) predicted that GATA5 was positively correlated with ARHGAP9. To verify the interaction between GATA5 and ARHGAP9, luciferase report assay was performed on the Luciferase Reporter System (Promega, USA). ARHGAP9 wild-type (WT) and mutant (MUT) were constructed and cloned into the luciferase vector. GATA5 mimic and mimic NC were transfected into A549 cells strictly in line with manufactureʹs specification. Finally, the luciferase activity was measured using Dual Luciferase Reporter Assay System (Promega, USA).

### Chromatin immunoprecipitation (ChIP) assay

To confirm the binding of GATA5 and ARHGAP9 promoters, A549 cells were scraped into 1× PBS and centrifuged at 800 × g for 5 min. Re-suspended with SDS lysis buffer for 15 min on ice, the pellets were centrifuged at 800 × g for 5 min and resuspended in nuclear lysis buffer. Subsequently, GATA5 antibody was incubated with protein A agarose beads (Santa Cruz, USA) and cell lysates at 4°C overnight. At last, the mRNA expression of TRIB3 was detected using RT-qPCR.

### Statistical analysis

All data collected from experiments were displayed as mean ± standard deviation and assessed with GraphPad Prism 8.0 software (GraphPad Software, Inc.). The statistical significance of differences among groups was analyzed with the application of Tukeyʹs test and one-way analysis of variance (ANOVA). Comparisons among two groups were analyzed using an unpaired studentʹs t-test. P less than 0.05 was viewed to show statistical significance.

## Results

### ARHGAP9 was downregulated in lung cancer and had relation with poor prognosis

**Figure 1.**
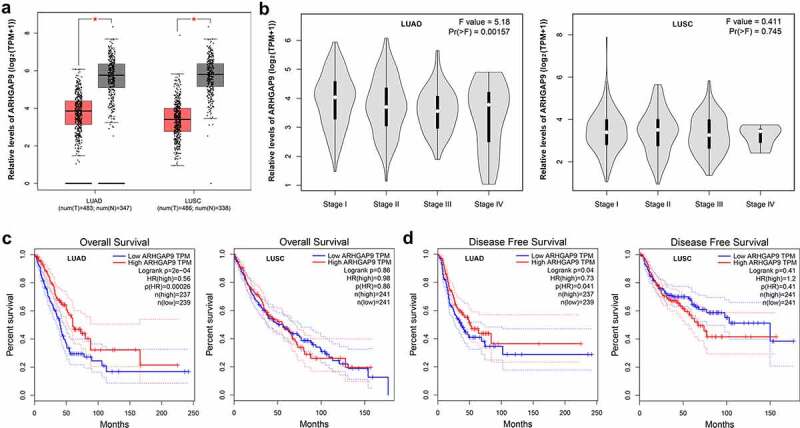
Expression of ARHGAP9 was analyzed by GEPIA database. (a) ARHGAP was downregulated in in non-small cell lung cancer patients. (b) The low expression of ARHGAP9 was significantly correlated with the pathological stage of lung adenocarcinoma. (c) The low expression of ARHGAP9 was significantly correlated with the low overall survival rate of lung adenocarcinoma patients. (d) The low expression of ARHGAP9 was significantly correlated with the low disease-free survival rate of lung adenocarcinoma patients. LUAD, lung adenocarcinoma; LUSC, lung squamous cell carcinoma.

Expression of ARHGAP9 in lung cancer was analyzed by GEPIA database. ARHGAP9 was downregulated in patients suffering from lung carcinoma (Figure 1(a)). Elsewhere, it was found that the ARHGAP9 expression was significantly correlated with the pathological phase of lung adenocarcinoma (Figure 1(b)). Results from GEPIA database also showed that ARHGAP9 downregulation was related to the low overall survival rate of lung adenocarcinoma patients (Figure 1(c)). Notably, the low expression of ARHGAP9 had close relation with the low disease-free survival rate of lung adenocarcinoma patients (Figure 1(d)).

### ARHGAP9 overexpression inhibited the proliferation of lung carcinoma cells

**Figure 2.**
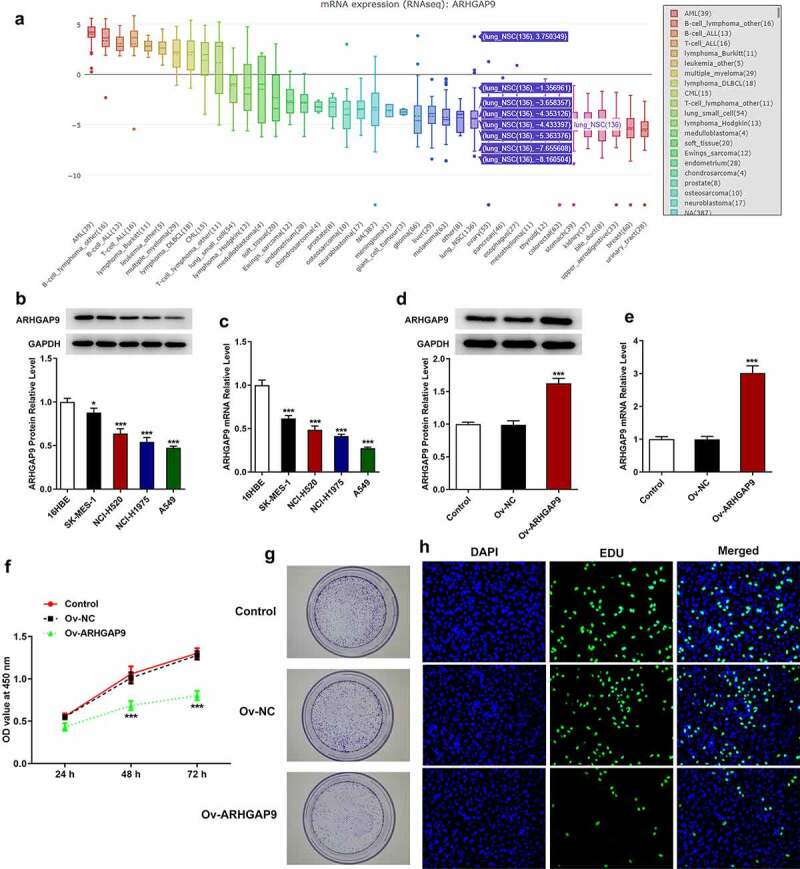
ARHGAP9 overexpression inhibited the proliferation of lung adenocarcinoma cells. (a) ARHGAP9 had a low expression in non-small cell lung cancer cell lines analyzed by CCLE database. The protein (b) and mRNA (c) expressions of ARHGAP9 were measured with Western blot and RT-qPCR. *P < 0.05 and ***p < 0.001 vs 16HBE. The protein (d) and mRNA (e) expressions of ARHGAP9 after transfection were measured with Western blot and RT-qPCR. (f) The A549 cell viability was detected by using CCK-8. (g) The proliferation and tumorigenesis ability of A549 cells were detected using colony formation assay (magnification, x100). (h) The proliferation of A549 cells was detected by using EDU staining (magnification, x200). ***p < 0.001 vs Ov-NC.

According to CCLE database (https://sites.broadinstitute.org/ccle), ARHGAP9 had a low expression in non-small-cell lung carcinoma patients (Figure 2(a)). As Figure 2(b,c) depicted, the protein and mRNA expressions of ARHGAP9 were greatly reduced in non-small-cell lung carcinoma cells, especially in A549 cells, in view of this, we selected A549 cells for the following experiments. In order to overexpress ARHGAP9, overexpression ARHGAP9 plasmids were utilized to transfect into A549 cells. Results from Figure 2(d,e) revealed that the protein and mRNA expressions of ARHGAP9 gained a huge growth in ARHGAP9-overexpressed A549 cells compared with negative control (Ov-NC).

Cell growth and proliferation were sequentially analyzed by CCK-8, cell cloning, and EDU staining assay. Compared with Ov-NC, the viability of A549 cells was tremendously decreased (Figure 2(f)). In addition, as Figure 2(g) suggested, the increased proliferation ability was significantly diminished after transfection with overexpression ARHGAP9 plasmids compared with Ov-NC. Likewise, results obtained from EDU staining further demonstrated that ARHGAP9 overexpression exhibited inhibitory effects on the proliferation of A549 cells, evidenced by the decreased proliferation in Ov-ARHGAP9 (Figure 2(h)).

### ARHGAP9 overexpression inhibited the metastasis of lung adenocarcinoma cells

**Figure 3.**
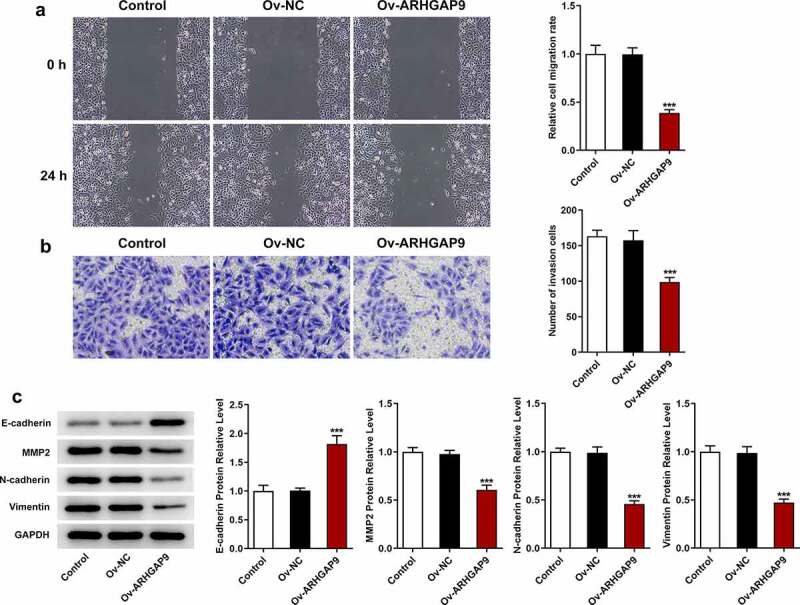
ARHGAP9 overexpression inhibited the invasive and migration of lung adenocarcinoma cells. (a) The relative cell migration rate was detected using wound healing assay (magnification, x100). (b) The number of invasion cells was detected using transwell (magnification, x100). (c) The expressions of migration-related proteins were measured by using Western blot, including E-cadherin, MMP2, N-cadherin and vimentin. ***p < 0.001 vs Ov-NC.

The migration and invasion of A549 cells were assessed with wound healing and transwell, respectively. According to Figure 3(a), the relative cell migration rate of Ov-ARHGAP9 was significantly lower than that of Ov-NC. Furthermore, the number of invasion cells was also decreased by ARHGAP9 overexpression in comparison with Ov-NC (Figure 3(b)). Besides, the expressions of metastasis-related proteins, including E-cadherin, MMP2, N-cadherin and Vimentin, were measured with Western blot. As Figure 3(c) depicted, overexpression ARHGAP9 upregulated E-cadherin expression but downregulated the expressions of MMP2, N-cadherin and Vimentin in contrast with Ov-NC.

### GATA5 transcription activated ARHGAP9

**Figure 4.**
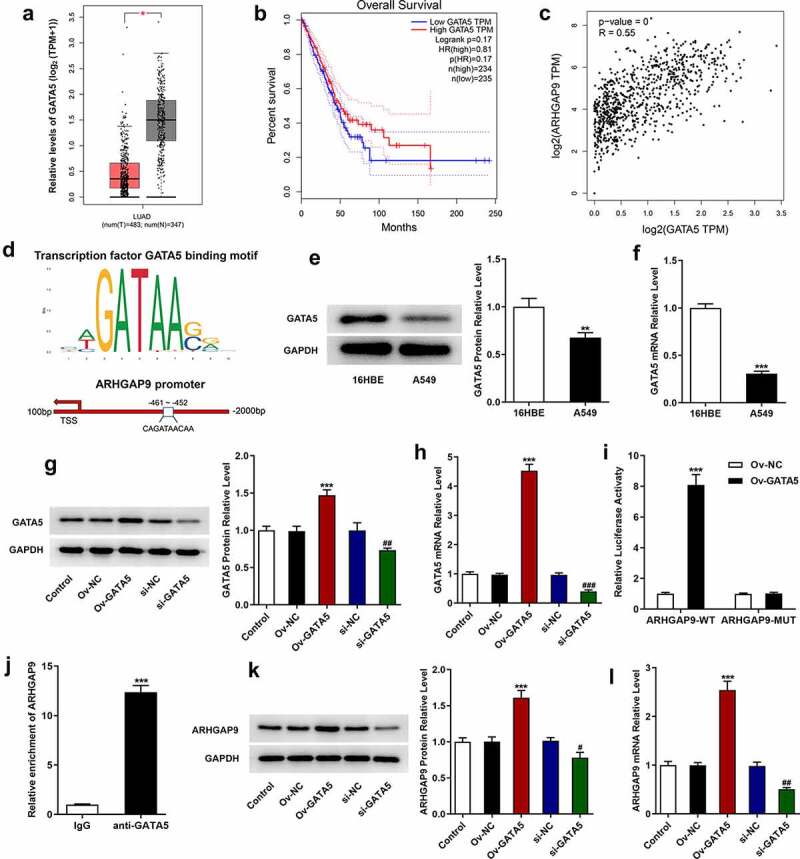
GATA5 and ARHGAP9 were positively correlated. (a) GATA5 was low expression in LUAD analyzed by GEPIA database. (b) Relationship between GATA5 and overall survival rate of lung adenocarcinoma patients analyzed by GEPIA database. (c) GATA5 was positively correlated with ARHGAP9 expression in lung adenocarcinoma tissues analyzed by GEPIA database. (d) The binding of GATA5 and ARHGAP9 promoters predicted by JASPAR database. The protein (e) and mRNA (f) expressions of GATA5 in lung adenocarcinoma cell lines were measured with Western blot and RT-qPCR. **P < 0.01 and ***p < 0.001 vs 16HBE. The protein (g) and mRNA (h) expressions of GATA5 after transfection were measured with Western blot and RT-qPCR. ***p < 0.001 vs Ov-NC, ^##^P < 0.01 and ^###^P < 0.001 vs si-NC. (i) Relative luciferase activity of A549 cells was evaluated using luciferase report. ***p < 0.001 vs ARHGAP9-WT + Ov-GATA5. (j) The relative enrichment of ARHGAP9 was assessed using ChIP. ***p < 0.001 vs IgG. The protein (k) and mRNA (l) expressions of ARHGAP9 were measured with Western blot and RT-qPCR. ***p < 0.001 vs Ov-NC, ^#^P < 0.5 and ^##^P < 0.01 vs si-NC.

GATA5 expression was analyzed by the GEPIA database and the binding site for GATA5 and ARHGAP9 was predicted using the JASPAR database. To figure out the mRNA and protein expressions of GATA5 in lung adenocarcinoma cells, RT-qPCR and Western blot were adopted. According to GEPIA database, GATA5 had a low expression in lung adenocarcinoma (Figure 4(a)). However, GATA5 downregulation had no relation with low overall survival rate of lung adenocarcinoma patients (Figure 4(b)). Besides, it is noted that GATA5 was positively correlated with ARHGAP9 expression in lung adenocarcinoma (Figure 4(c)). Moreover, JASPAR database predicted the binding of GATA5 and ARHGAP9 promoters (Figure 4(d)). As Figure 4(e,f) shown, GATA5 expression was significantly downregulated in A549 cells in comparison with 16HBE cells. Compared with Ov-NC, GATA5 gained a huge growth in GATA5-overexpressed A549 cells. Besides, GATA5 silence brought about the decreased GATA5 expression in A549 cells (Figure 4(g,h)).

Luciferase report gene assay verified that GATA5 overexpression enhanced the luciferase activity of ARHGAP9-WT but had no obvious promotive effects on ARHGAP9-MUT (Figure 4(i)). Additionally, ChIP was also adopted to detect the binding ability of GATA5 and ARHGAP9. It is noted that relative enrichment of ARHGAP9 in anti-GATA5 was significantly higher than that in IgG (Figure 4(j)). Moreover, GATA5 overexpression upregulated ARHGAP9 expression and GATA5 silence downregulated ARHGAP9 expression in comparison with control group (Figure 4(k,l)).

### GATA5 reversed the inhibitory effects of ARHGAP9 overexpression on the proliferation, invasion and migration in lung adenocarcinoma cells

**Figure 5.**
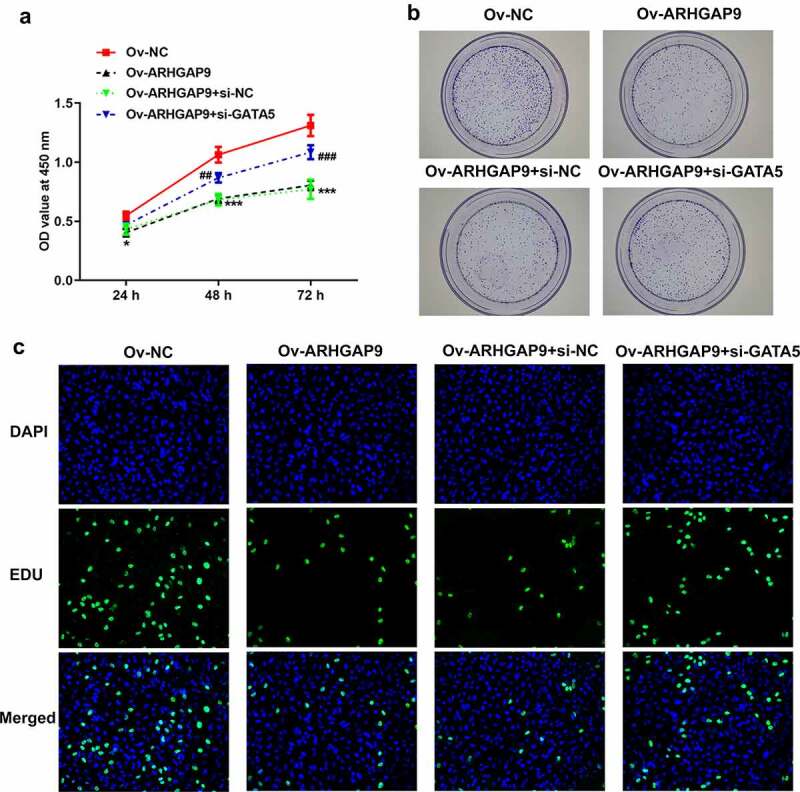
GATA5 reversed the inhibitory effects of ARHGAP9 overexpression on the proliferation in lung adenocarcinoma cells. (a) The viability of A549 cells was detected using CCK-8. ***p < 0.001 vs Ov-NC; ^##^P < 0.01 and ^###^P < 0.001 vs Ov-ARHGAP9 + si-NC. (b) The proliferation and tumorigenesis ability of A549 cells were detected using colony formation assay (magnification, x100). (c) The proliferation of A549 cells was detected using EDU staining (magnification, x200).

**Figure 6.**
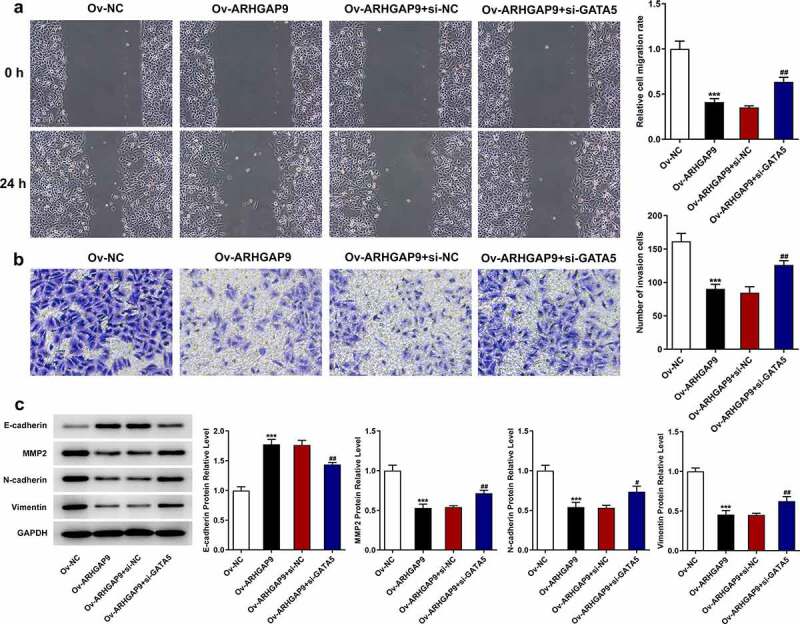
GATA5 reversed the inhibitory effects of ARHGAP9 overexpression on the invasion and migration in lung adenocarcinoma cells. (a) The relative cell migration rate of A549 cells was detected using wound healing assay (magnification, x100). (b) The number of invasion cells was detected using transwell (magnification, x100). (c) The expressions of migration-related proteins were measured using Western blot, including E-cadherin, MMP2, N-cadherin and vimentin. ***p < 0.001 vs Ov-NC, ^#^P < 0.5 and ^##^P < 0.01 vs Ov-ARHGAP9 + si-NC.

The study co-transfected Ov-ARHGAP9 and si-GATA5 into cells and repeated the above experiments to further investigate the relationship between ARHGAP9 and GATA5. In comparison with Ov-NC, ARHGAP9 overexpression significantly decreased the viability of A549 cells. However, GATA5 silence partially reversed the viability of A549 cells, evidenced by the increased cell viability in Ov-ARHGAP9 + si-GATA (Figure 5(a)). The decreased cell proliferation and tumorigenesis ability caused by ARHGAP9 overexpression were then enhanced in Ov-ARHGAP9 + si-GATA5 (Figure 5(b)). Likewise, ARHGAP9 overexpression reduced the cell proliferation while GATA5 silence reversed the inhibitory effects of ARHGAP9 overexpression, evidenced by the increased proliferation in comparison with Ov-ARHGAP9 + si-NC (Figure 5(c)). In addition, it was found that ARHGAP9 overexpression tremendously diminished the relative cell migration rate. However, compared with Ov-ARHGAP9 + si-NC, the decreased cell migration rate was partially increased by GATA5 silence (Figure 6(a)). Moreover, the diminished number of invasion cells caused by ARHGAP9 overexpression was increased after transfection with GATA5 silence plasmids (Figure 6(b)). As Figure 6(c) depicted, the upregulation of E-cadherin expression and downregulation of MMP2, N-cadherin and vimentin expression by overexpression of ARHGAP9 was reversed by silencing of GATA5 (Ov-ARHGAP9 + si-GATA5 vs. Ov-ARHGAP9 + si-NC).

## Discussion

Lung cancer, the most frequently diagnosed cancer type, is the top-ranked contributor to the cancer death worldwide [2]. Lung adenocarcinoma, which takes up over 40% of lung cancer, is the most common subtype of lung cancer [19]. Although great progresses have been made in treatment strategies, including molecular targeted drugs and immune checkpoint inhibitors, the average 5-year relative survival rate of patients living with lung cancer still remains low [20]. The present study found that ARHGAP9 was low expression in lung adenocarcinoma and had relation with poor prognosis. ARHGAP9 upregulation inhibited the malignant progression of lung adenocarcinoma cells. Furthermore, GATA5 effectively activated ARHGAP9. Whatʹs more, GATA5 silencing reversed the inhibitory effects of ARHGAP9 overexpression on the proliferation, invasion and migration in lung adenocarcinoma cells.

ARHGAP9, also known as RhoGAP9 [7], was testified to be a cancer-associated gene [21]. Researchers held the opinion that ARHGAP9 expression had close relation with poor patient survival and its upregulation exhibited suppressive effects on the invasion, proliferation and migration of liver cancer cells [14]. Additionally, ARHGAP9 was supposed to be a useful marker for the treatment of bladder cancer as its downregulated was correlated with bladder prognosis, and the study found a nearly 3-fold increased risk of cancer-specific death when ARHGAP9 levels were reduced [22]. Based on statistical analysis in the GEPIA database, ARHGAP9 expression was found to be significantly lower in lung cancer tissue than in paired normal tissues. Subsequently, we further investigated the role of ARHGAP9 in lung adenocarcinoma by transfecting overexpressed ARHGAP9 plasmids into lung adenocarcinoma cells. The results of the present study were consistent with expectations, when ARHGAP9 was upregulated, lung adenocarcinoma cell proliferation was suppressed significantly in comparison with the control group, while the invasion and migration ability of cancer cells were diminished.

Of interest, the study demonstrated that reduced and/or absent expression of E-cadherin in non-small cell lung cancer was responsible for the malignant phenotype, and moreover, aberrant expression of N-cadherin was closely associated with transformation, adhesion, apoptosis, angiogenesis, and invasive metastasis in human malignancies [23,24]. Therefore, in the present study, we performed an analysis of E-cadherin and N-cadherin protein expression and found that upregulation of ARHGAP9 clearly promoted E-cadherin and inhibited N-cadherin expression comparing to the control group.

To complete the study, the analysis of the regulation of ARHGAP9 upstream factors was performed by introducing GATA5. GATA5 is a transcription factor that inhibits the development of various human cancer types [17]. Research has revealed that GATA5 deficiency might induce airway constrictor hyperresponsiveness by attenuating apolipoprotein E and increasing IL-13 expression [25]. In this study, we discovered that GATA5 was downregulated in lung adenocarcinoma tissues and positively correlated with ARHGAP9 in lung adenocarcinoma. Notably, regulation of GATA5 was found to directly affect the mRNA and protein expression of ARHGAP9 in lung adenocarcinoma cells. And the suppressive effects of ARHGAP9 overexpression on proliferation, invasion and migration in lung adenocarcinoma were reversed by GATA5 silencing. Thus, the study successfully demonstrated that GATA5 could positively induce ARHGAP9 to inhibit lung adenocarcinoma cell progression.

## Conclusion

The study found that ARHGAP9 upregulation had a significant inhibitory effect on proliferation, invasion and migration of lung cancer cells. In addition, ARHGAP9 expression could be cis-regulated by GATA5. Thus, the present study was the first to suggest a protective effect of GATA5 induced ARHGAP9 on lung adenocarcinoma cells. Also, to validate the present findings, future studies will be conducted in in vivo models and clinical trials targeting ARHGAP9 as a biomarker.

## Highlights

1. GATA5 activated ARHGAP9 in lung adenocarcinoma cells;
2. ARHGAP9 upregulation significantly inhibited lung adenocarcinoma cell proliferation, invasion and migration;
3. GATA5-induced ARHGAP9 upregulation had an inhibitory effect on the malignant progression of lung adenocarcinoma cells.
